# Simultaneous detection of *Helicobacter pylori* infection comparing between white light and image-enhanced endoscopy

**DOI:** 10.1186/s12876-024-03132-y

**Published:** 2024-01-26

**Authors:** Boonyaorn Chatrangsun, Natsuda Aumpan, Bubpha Pornthisarn, Soonthorn Chonprasertsuk, Sith Siramolpiwat, Patommatat Bhanthumkomol, Pongjarat Nunanan, Navapan Issariyakulkarn, Varocha Mahachai, Yoshio Yamaoka, Ratha-korn Vilaichone

**Affiliations:** 1 Department of Medicine, Lampang hospital, Lampang, Thailand; 2 Center of Excellence in Digestive Diseases and Gastroenterology Unit, Department of Medicine, Thammasat University, Pathumthani, Thailand; 3 Department of Medicine, Chulabhorn International College of Medicine (CICM) at Thammasat University, Pathumthani, Thailand; 4 Department of Environmental and Preventive Medicine, Oita University Faculty of Medicine, Yufu, Japan; 5 Research Center for Global and Local Infectious Diseases, Oita University, Yufu, Japan; 6 Department of Medicine, Gastroenterology and Hepatology Section, Baylor College of Medicine, Houston, TX USA

**Keywords:** *Helicobacter pylori*, Diagnosis, Image-enhanced endoscopy

## Abstract

**Background:**

*Helicobacter pylori* (*H. pylori*) is associated with gastric cancer. Early and accurate diagnosis of *H. pylori* infection can reduce risk of gastric cancer. Conventional white light imaging (WLI) and image-enhanced endoscopic (IEE) techniques such as narrow-band imaging (NBI), linked color imaging (LCI) and blue laser imaging (BLI) plays pivotal role in *H. pylori* diagnosis. This study aimed to determine diagnostic performance of real-time endoscopy between WLI and other IEE techniques for diagnosis of *H. pylori* infection.

**Methods:**

This prospective study compared endoscopic images by gastroscopy using WLI and IEE techniques (LCI, Magnifying-BLI, and Magnifying-NBI) at Thammasat University Hospital, Thailand between January 2020, and July 2021. All participants underwent gastroscopy. Three biopsies at gastric antrum and two biopsies at body were obtained for *H.pylori* diagnosis. *H. pylori* infection was defined as a positive test of either one of the following tests: rapid urease test, histopathology, *H. pylori* culture.

**Results:**

Of 167 dyspeptic patients undergoing gastroscopy, 100 were enrolled in this study. Overall *H. pylori* infection was 40%. Patients had the mean age of 59.1 years and 53% were males. Enlarged gastric folds and antral nodularity can predict *H. pylori* infection with 100% PPV, while fundic gland polyps and red streak provided 100% PPV for exclusion of *H. pylori* infection on WLI. Sensitivity, specificity, PPV, NPV and accuracy for diagnosis of *H. pylori* infection for WLI were 80%, 71.7%, 65.3%, 84.3% and 75% respectively, while those for LCI were 90%, 70%, 66.7%, 91.3% and 78% respectively. M-NBI and M-BLI endoscopy demonstrated elongated pits in *H. pylori*-positive patients. Sensitivity, specificity, PPV, NPV and accuracy for M-BLI were 95%, 80%, 76%, 96% and 86% respectively, whereas those for M-NBI were 92.5%, 86.7%, 82.2%, 94.6% and 89% respectively. Sensitivity of M-BLI was better than WLI, while sensitivities of LCI and M-NBI were also numerically higher than WLI without statistical difference (M-BLI 95%vs.WLI 80%, *p* = 0.03; M-NBI 92.5%vs.WLI 80%, *p* = 0.13; LCI 90%vs.WLI 80%, *p* = 0.22). Sensitivities of all IEE modes were not different from one another (LCI 90%vs.M-BLI 95%, *p* = 0.50; LCI 90%vs.M-NBI 92.5%, *p* = 1.00, M-BLI 95%vs.M-NBI 92.5%, *p* = 1.00).

**Conclusions:**

M-BLI significantly improved sensitivity of real-time endoscopic diagnosis of *H. pylori* infection compared with WLI. Enlarged gastric folds and antral nodularity could be reliable predictors for *H. pylori* infection, while fundic gland polyps and red streak could be important endoscopic findings for *H. pylori*-negative mucosa.

**Supplementary Information:**

The online version contains supplementary material available at 10.1186/s12876-024-03132-y.

## Introduction

*Helicobacter pylori* (*H. pylori*), a spiral-shaped Gram-negative bacterium, is a cause of common persistent infection worldwide [1]. After adherence to gastric epithelial cells, *H. pylori* induces chronic gastritis which can result in more severe conditions such as peptic ulcers, gastric mucosa-associated lymphoid tissue lymphoma, and particularly gastric adenocarcinoma through the Correa’s cascade [2, 3]. Classified as a definite carcinogen, *H. pylori* eradication is recommended to prevent gastric cancer [4, 5]. Early and accurate diagnosis of *H. pylori* infection is an initial step for gastric cancer prevention. Although non-invasive tests can be used to detect *H. pylori* infection in young patients without alarm features [2], upper gastrointestinal (GI) endoscopy is indicated in dyspeptic patients with alarm symptoms, age of onset after 50 years, and non-responders to proton pump inhibitor (PPI) trial [6]. Several endoscopy-based tests require biopsy samples resulting in mucosal injury and high medical expenses. Recognition of specific endoscopic findings according to the Kyoto classification of gastritis can help physicians determine *H. pylori* infection status and avoid unnecessary biopsies [7].

Image-enhanced endoscopy (IEE) is a diagnostic modality developed to enhance contrast of mucosal surface and provide more accurate diagnosis of gastric lesions [8]. There are various IEE techniques including narrow band imaging (NBI), blue laser imaging (BLI), and linked color imaging (LCI) [8, 9]. NBI and BLI use specific bands of light which are absorbed by hemoglobin in blood vessels but reflected by surrounding mucosa to obtain detailed images of microvasculature and microstructure on the mucosal surface [10, 11]. LCI displays brighter mucosal color than conventional white light imaging (WLI) and can be used as a screening tool for gastrointestinal lesions [11]. IEE provides high diagnostic performance in detection of dysplasia and early gastric cancer as recommended by the recent guideline [2]. However, real-time endoscopic diagnosis of *H. pylori* infection by IEE were reported in limited studies [9, 12]. The previous meta-analysis suggested that assessment of pit and vascular patterns in the gastric body could also provide high diagnostic potential for *H. pylori* infection [13].

Although there has been more popular use of IEE for diagnosis of gastric precancerous lesions and *H. pylori* infection in the past decade, most studies were from East Asian countries. Until now, there has been limited data about diagnostic performance of IEE on *H. pylori* infection in Thailand. This study aimed to determine diagnostic performance of different IEE modalities for *H. pylori* infection.

## Methods

### Study design

This was a prospective study conducted at tertiary care center in Thailand between January 2020 and July 2021. The inclusion criteria were Thai patients aged 18–75 years who underwent esophagogastroduodenoscopy (EGD) as indicated for diagnostic evaluation of symptoms (e.g., dyspepsia, dysphagia, chronic abdominal pain, and iron deficiency anemia). The exclusion criteria were patients with severe comorbidities, contraindication to gastric biopsy, upper GI bleeding, previous *H. pylori* eradication, pregnancy, or unwilling to participate in the study. Informed consent was obtained from all patients prior to the enrolment and all study procedures.

### Endoscopic procedure

All endoscopic examinations were performed by three expert endoscopists who had previously performed > 1,000 EGDs. EGD was performed using a GIF-290 endoscope (Olympus Co. Ltd., Tokyo, Japan) and an EG-760R endoscope (Fujifilm Co., Tokyo, Japan). Every patient underwent EGD by both endoscopes. The first endoscope was randomly assigned and inserted into the stomach. Adequate air insufflation and application of mucosal cleaning techniques were used to achieve satisfactory endoscopic visualization [14]. Endoscopic image documentation of anatomical landmarks of the stomach was performed by capturing at least 4 representative photographs using conventional WLI: (1) gastric cardia and fundus on retroflexed view, (2) gastric body in either retroflexed or forward view, (3) incisura angularis on retroflexed view, (4) antrum and pylorus. If other obvious lesions such as erosion, or ulcer, or polyp were seen, additional photography of these lesions were performed. Subsequently, the endoscopist activated LCI, BLI, or NBI modes. Image documentation in LCI mode was similar to WLI, whereas images in NBI or BLI mode were magnified and captured in close-up views at greater curvature of mid gastric body to carefully examine gastric mucosal architecture and vascular patterns. Patients subsequently underwent EGD by the second endoscope and the endoscopist activated the remaining IEE mode to record mucosal patterns. The number of endoscopic images for diagnosis of *H. pylori* infection were at least 4, 4, 1, and 1 for WLI, LCI, BLI, and NBI, respectively. After gastric images of all patients had been collected, endoscopic *H. pylori* infection status of each patient was determined by the consensus of three expert endoscopists who were blinded to results of rapid urease test, histopathology, and culture. The criteria for endoscopic diagnosis of *H. pylori* infection by each IEE mode is in the next subsection.

### Endoscopic diagnosis of ***H. pylori*** infection

Normal gastric mucosa without *H. pylori* infection can be determined by various endoscopic findings. The presence of a regular arrangement of collecting venules (RAC) at lesser curvature on WLI is a predictor of *H. pylori*-negative stomach with high sensitivity [15]. RAC is a tiny red starfish-like appearance regularly distributed throughout the entire lesser curvature [15]. LCI demonstrated white apricot mucosa in *H. pylori*-negative patients [16], while magnifying NBI (M-NBI) or magnifying BLI (M-BLI) showed small round pits encircled by regular honeycomb-like subepithelial capillary networks (SECNs) and interspersed collecting venules in the gastric body [12, 17].

Endoscopic features on WLI associated with *H. pylori* infection are diffuse redness, spotty redness, antral nodularity, sticky mucus, enlarged gastric folds, mucosal edema, xanthoma, and hyperplastic polyp [18]. LCI can enhance color contrast and improve endoscopic detection of *H. pylori* infection. Deep crimson mucosa of entire fundic gland region and spotty redness by LCI were demonstrated in *H. pylori*-positive patients [9, 19]. NBI system uses filters to produce 415-nm blue and 540-nm green light which are absorbed by hemoglobin in superficial capillaries and deeper submucosal vessels, respectively [10]. These bands of light are reflected by mucosa creating contrast between vascular structures and surrounding mucosa [10]. BLI uses 410-nm blue-violet and 450-nm blue lasers to obtain detailed images of microstructure on the mucosal surface [11]. In *H. pylori*-positive patients, M-NBI or M-BLI revealed enlarged or elongated pits with obscure SECNs or dense fine irregular vessels [12, 20]. Data of endoscopic features were collected according to each endoscopic mode as follows:

1. Endoscopic diagnosis of *H. pylori* infection on **WLI** or **LCI** was defined as comprehensive findings on WLI or LCI which were the presence of at least one of eight endoscopic findings based on Kyoto classification: diffuse redness, spotty redness, antral nodularity, sticky mucus, enlarged gastric folds, mucosal edema, xanthoma, or hyperplastic polyp.
2. Endoscopic diagnosis of *H. pylori* infection on **M-BLI** or **M-NBI** was defined as the presence of enlarged or elongated pits with obscure SECNs or dense fine irregular vessels.

### Detection of ***H. pylori*** infection

After image documentation by the second endoscopy, five gastric biopsies (3 biopsies at antrum and 2 biopsies at body) were obtained. Rapid urease test (one biopsy from antrum and one from gastric body), histopathology (one biopsy from antrum and one from gastric body), and *H. pylori* culture (one biopsy from antrum) were performed in all patients. *H. pylori* infection was defined as a positive test of either one of the aforementioned tests.

### Statistical analysis

All data were analyzed by using SPSS version 22 (SPSS Inc., Chicago, IL, USA). Categorical variables were analyzed by Fisher’s exact test, or Chi-squared test where appropriate. Continuous variables were analyzed by using Student’s t-test. For endoscopic mucosal pattern evaluation, sensitivities, specificities, positive predictive values (PPV), negative predictive values (NPV), and diagnostic accuracies were calculated for each endoscopic technique. Comparison of sensitivities between diagnostic methods was analyzed by McNemar’s test with Yates’ continuity correction. The p-value < 0.05 was considered as statistical significance. This study was approved by the Human Research Ethics Committee of Thammasat University (MTU-EC-IM-6-196/62).

## Results

**Fig. 1:**
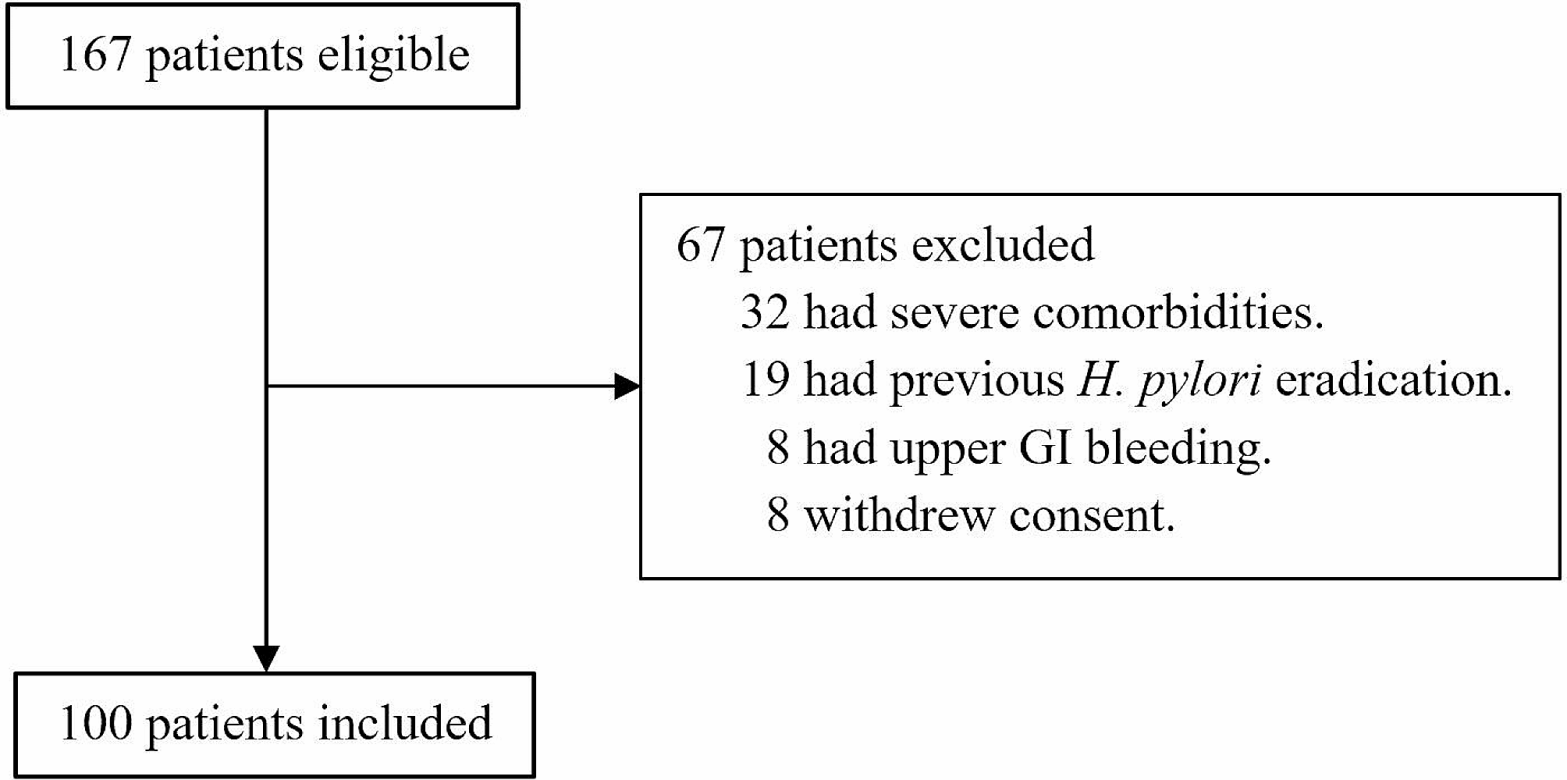
Patient enrollment flowchart

Of 167 patients enrolled in this study, 32 patients with severe comorbidities, 19 with previous *H. pylori* eradication, 8 with upper GI bleeding, and 8 who withdrew informed consent were excluded (Fig. 1). One hundred patients were included in this study with the mean age of 59.1 ± 11.5 years and 53% were men. All patients had indications for upper GI endoscopy suggested by the Gastroenterological Association of Thailand. Indications were dyspepsia (46%), chronic abdominal pain (30%), iron deficiency anemia (16%), and dysphagia (8%). The prevalence of *H. pylori* infection was 40%. Majority of patients had no comorbidity (53%). Hypertension (33%) was the most common comorbidity, followed by dyslipidemia (30%), and diabetes mellitus (22%) as demonstrated in Table 1. Endoscopic findings demonstrated peptic ulcers (17%: gastric ulcer 13%, gastric and duodenal ulcers 3%, duodenal ulcer 1%,), intestinal metaplasia (17%), and gastric atrophy (8%).

**Table 1:** Baseline characteristics of patients

Factors	Total patients (*N* = 100)
Gender (%Male)	53 (53%)
Age (years ± SD)	59.1 ± 11.5
Underlying diseases	
None	53 (53%)
Hypertension	33 (33%)
Dyslipidemia	30 (30%)
Diabetes mellitus	22 (22%)
Cirrhosis	20 (20%)
Medication use	
NSAID	16 (16%)
Aspirin	14 (14%)
Alcohol use	18 (18%)
Smoking	9 (9%)
Endoscopic findings	
Peptic ulcers	17 (17%)
Intestinal metaplasia	17 (17%)
Gastric atrophy	8 (8%)

NSAID = Nonsteroidal anti-inflammatory drug

### Diagnostic performance of endoscopic modalities for diagnosis of ***H. pylori*** infection

**Fig. 2:**
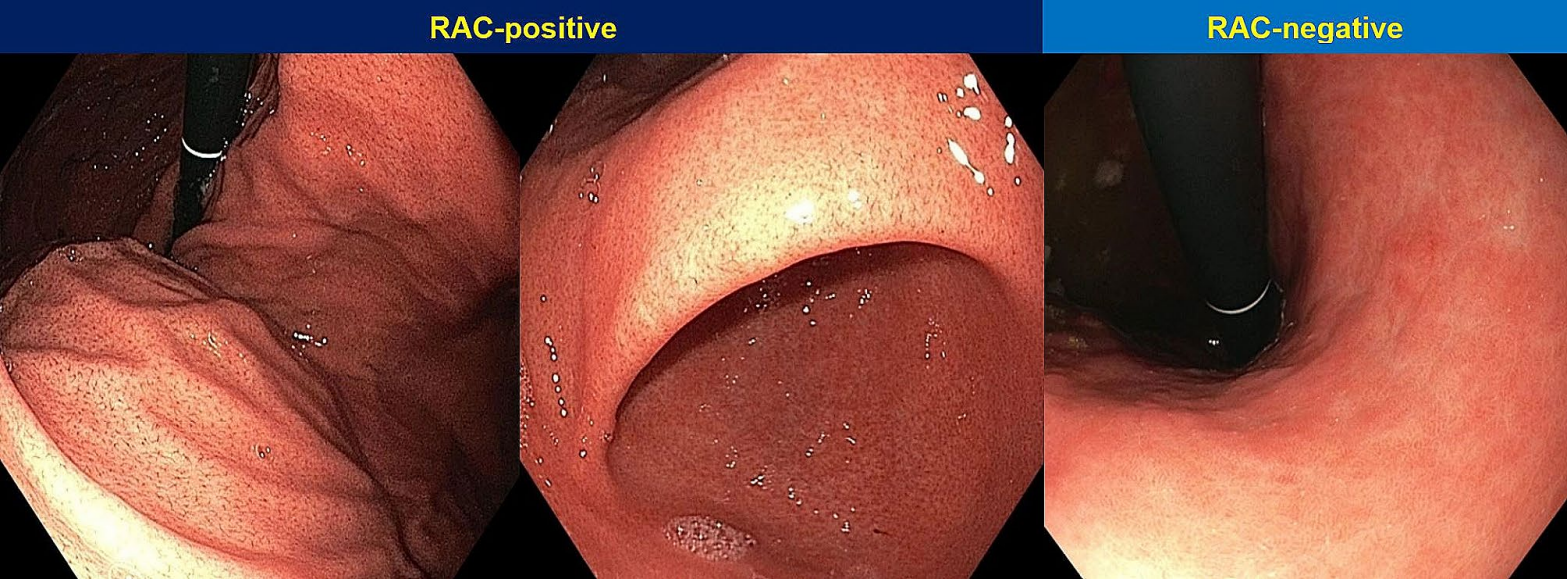
RAC-positive and RAC-negative pattern on WLI

Several endoscopic features associated with *H. pylori* infection were determined by WLI. Mucosal edema, diffuse redness, spotty redness, sticky mucus, enlarged gastric folds, antral nodularity, gastric xanthoma, and hyperplastic polyp on WLI were related to *H. pylori* infection with sensitivity/specificity of 75%/78.3%, 57.5%/90%, 35%/95%, 12.5%/98.3%, 12.5%/100%, 10%/100%, 2.5%/98.3%, and 0%/98.3%, respectively. Mucosal edema provided the highest sensitivity to diagnose *H. pylori* infection on WLI (75%). However, false positive rate was high (21.7%), especially in cirrhotic patients since 28.6% of them with mucosal edema did not have *H. pylori* infection. Enlarged gastric folds and antral nodularity on WLI exhibited 100% PPV for diagnosis of *H. pylori* infection. Fundic gland polyps, red streak, and presence of RAC on WLI demonstrated excellent PPV for predicting *H. pylori*-negative status of 100%, 100%, and 96.3%, respectively. RAC-positive and RAC-negative patterns on WLI were presented in Fig. 2. Sensitivities/specificities of mucosal edema, diffuse redness, spotty redness, sticky mucus, enlarged gastric folds, antral nodularity, gastric xanthoma, and hyperplastic polyp on LCI for diagnosis of *H. pylori* infection were 80%/80%, 75%/90%, 55%/95%, 10%/98.3%, 12.5%/100%, 12.5%/100%, 2.5%/96.7%, and 0%/98.3%, respectively. When LCI mode was activated, diffuse redness and spotty redness were more commonly detected by color enhancement. Sensitivities of diffuse redness and spotty redness for diagnosis of *H. pylori* infection using LCI mode increased by 30.4% and 57.1% compared with WLI, respectively. Diagnostic performance of endoscopic findings for diagnosis of *H. pylori* status by WLI or LCI was demonstrated in Table 2.

**Table 2:** Diagnostic performance of endoscopic findings for diagnosis of *H. pylori* status by WLI or LCI

**Diagnostic performance to predict** ***H. pylori-*positive status**						
**Findings on each endoscopic mode**		**Sensitivity**	**Specificity**	**PPV**	**NPV**	**Accuracy**
Mucosal edema	on WLI	75%	78.3%	69.8%	82.5%	77%
	on LCI	80%	80%	72.7%	85.7%	80%
Diffuse redness	on WLI	57.5%	90%	79.3%	76.1%	77%
	on LCI	75%	90%	83.3%	84.4%	84%
Spotty redness	on WLI	35%	95%	82.4%	68.7%	71%
	on LCI	55%	95%	88%	76%	79%
Sticky mucus	on WLI	12.5%	98.3%	83.3%	62.8%	64%
	on LCI	10%	98.3%	80%	62.1%	63%
Enlarged gastric folds	on WLI	12.5%	100%	100%	63.2%	65%
	on LCI	12.5%	100%	100%	63.2%	65%
Antral nodularity	on WLI	10%	100%	100%	62.5%	64%
	on LCI	12.5%	100%	100%	63.2%	65%
Gastric xanthoma	on WLI	2.5%	98.3%	50%	60.2%	60%
	on LCI	2.5%	96.7%	33.3%	59.8%	59%
Hyperplastic polyp	on WLI	0%	98.3%	0%	59.6%	59%
	on LCI	0%	98.3%	0%	59.6%	59%
**Diagnostic performance to predict** ***H. pylori*-negative status**						
**Findings on each endoscopic mode**		**Sensitivity**	**Specificity**	**PPV**	**NPV**	**Accuracy**
Presence of RAC	on WLI	86.7%	95%	96.3%	82.6%	90%
	on LCI	83.3%	85%	89.3%	77.3%	84%
Fundic gland polyps	on WLI	16.7%	100%	100%	55.6%	50%
	on LCI	16.7%	100%	100%	55.6%	50%
Hematin spots	on WLI	15%	97.5%	90%	56.7%	48%
	on LCI	16.7%	97.5%	90.9%	43.8%	51%
Red streak	on WLI	8.3%	100%	100%	42.1%	45%
	on LCI	8.3%	100%	100%	42.1%	45%

LCI = Linked Color Imaging, NPV = Negative predictive value, PPV = Positive predictive value, RAC = Regular arrangement of collecting venules, WLI = White Light Imaging

**Fig. 3:**
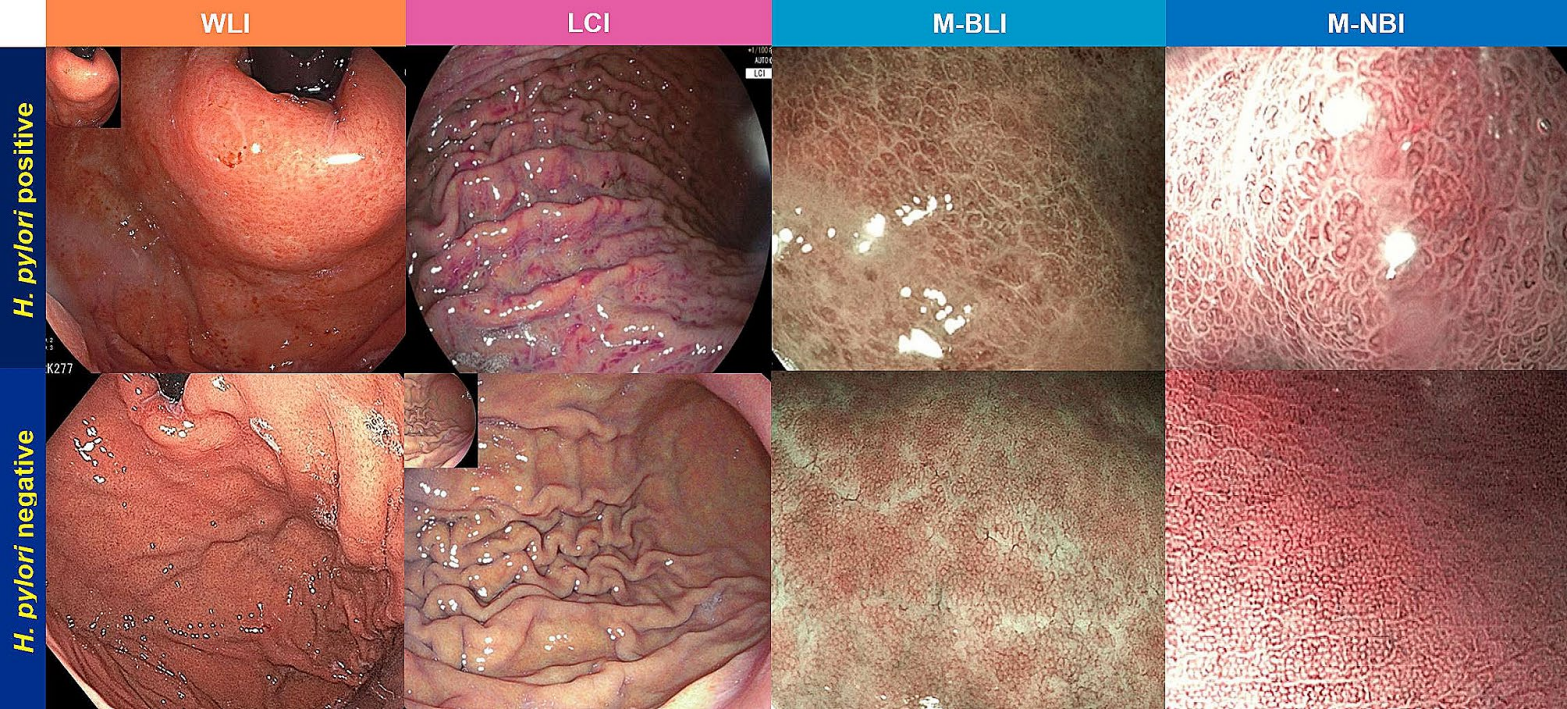
Endoscopic features of *H. pylori*-positive (upper row) and *H. pylori*-negative gastric mucosa (bottom row) by different endoscopic modalities **Upper row:** Spotty redness of mucosa was demonstrated in WLI and LCI, while M-BLI and M-NBI revealed elongated pits **Bottom row:** WLI showed normal mucosa, while LCI demonstrated white apricot mucosa. M-BLI and M-NBI showed small round pits at gastric body LCI = Linked Color Imaging, M-BLI = Magnifying Blue Light Imaging, M-NBI = Magnifying Narrow Band Imaging, WLI = White Light Imaging

WLI demonstrated sensitivity, specificity, PPV, NPV and accuracy of 80%, 71.7%, 65.3%, 84.3% and 75%, respectively for diagnosis of *H. pylori* infection. M-BLI mode reported the highest sensitivity of 95%, while specificity, PPV, NPV and accuracy were 80%, 76%, 96%, and 86%, respectively. Sensitivity, specificity, PPV, NPV and accuracy for M-NBI were 92.5%, 86.7%, 82.2%, 94.6% and 89% respectively, whereas those for LCI were 90%, 70%, 66.7%, 91.3% and 78% respectively. Diagnostic performance of each endoscopic modality for diagnosis of *H. pylori* infection was demonstrated in Table 3. Sensitivity of M-BLI was better than WLI, while sensitivities of LCI and M-NBI were also numerically higher than WLI without statistical difference (M-BLI 95% vs. WLI 80%, *p* = 0.03; M-NBI 92.5% vs. WLI 80%, *p* = 0.13; LCI 90% vs. WLI 80%, *p* = 0.22). Interestingly, there were 20% (8/40) of *H. pylori*-infected patients with endoscopically normal gastric mucosa on WLI, but most of them had their endoscopic *H. pylori* detection while using M-BLI (6/8) or M-NBI (6/8). Sensitivities of all IEE modes were not different from one another (LCI 90% vs. M-BLI 95%, *p* = 0.50; LCI 90% vs. M-NBI 92.5%, *p* = 1.00, M-BLI 95% vs. M-NBI 92.5%, *p* = 1.00). Different endoscopic features of *H. pylori*-positive and *H. pylori*-negative gastric mucosa by each endoscopic mode were demonstrated in Fig. 3.

**Table 3:** Diagnostic performance of each endoscopic modality for diagnosis of *H. pylori* infection

Endoscopic technique	Sensitivity (95% CI)	Specificity (95% CI)	PPV (95% CI)	NPV (95% CI)	Accuracy (95% CI)
**M-BLI** Enlarged or elongated pits with obscure SECNs or dense fine irregular vessels	95% (83.1–99.4)	80% (67.7–89.2)	76% (65.5–84.1)	96% (86.1–98.9)	86% (77.6–92.1)
**M-NBI** Enlarged or elongated pits with obscure SECNs or dense fine irregular vessels	92.5% (79.6–98.4)	86.7% (75.4–94.1)	82.2% (70.7–89.9)	94.6% (85.3–98.1)	89% (81.2–94.4)
**LCI** Comprehensive findings on LCI were the presence of ≥ 1 of 8 endoscopic findings based on Kyoto classification*	90% (76.3–97.2)	70% (56.8–81.2)	66.7% (57.3–74.9)	91.3% (80.3–96.4)	78% (68.6–85.7)
**WLI** Comprehensive findings on WLI were the presence of ≥ 1 of 8 endoscopic findings based on Kyoto classification*	80% (64.4–91.0)	71.7% (58.6–82.6)	65.3% (55.0-74.3)	84.3% (73.9–91.1)	75% (65.3–83.1)

LCI = Linked Color Imaging, M-BLI = Magnifying Blue Light Imaging, M-NBI = Magnifying Narrow Band Imaging, NPV = Negative predictive value, PPV = Positive predictive value, SECNs = Subepithelial capillary networks, WLI = White Light Imaging

*Eight endoscopic findings based on Kyoto classification included diffuse redness, spotty redness, antral nodularity, sticky mucus, enlarged gastric folds, mucosal edema, xanthoma, and hyperplastic polyp

## Discussion

Rapid and accurate diagnosis of *H. pylori* infection is essential for treatment initiation in order to prevent gastric cancer. This study highlighted the diagnostic performance of conventional WLI and IEE performed on the same patient for diagnosis of *H. pylori* infection. All IEE techniques improved sensitivities above 90% and might be used for real-time endoscopic diagnosis of *H. pylori* infection. Enlarged gastric folds and antral nodularity are endoscopic features for predicting *H. pylori* infection, whereas fundic gland polyps and red streak are demonstrated in *H. pylori*-negative gastric mucosa.

Endoscopic diagnosis of *H. pylori* infection can be initially determined by conventional WLI. This study demonstrated that mucosal edema could indicate *H. pylori* infection with the highest sensitivity on WLI (75%) which was comparable to the previous study (72.3%) [21]. However, we observed that false positive rate was high in cirrhotic patients with gastric mucosal edema as 28.6% of them did not have *H. pylori* infection. Therefore, mucosal edema should be interpreted cautiously in cirrhotic patients since it can be caused by congestion and hyperemia from portal hypertension itself [22]. Diffuse redness (57.5%) and spotty redness (35%) were identified in lower number of patients than prior studies (58–81% for diffuse redness, 60–65% for spotty redness) [21, 23]. Other features including sticky mucus, enlarged gastric folds, antral nodularity, and xanthoma demonstrated low sensitivities but high specificities (90–100%) for *H. pylori* diagnosis in this study. Fundic gland polyps and red streak could predict negative *H. pylori* status with 100% PPV which was higher than previous studies in China and Japan [7, 21, 24]. Presence of RAC yielded the highest sensitivity (86.7%) and accuracy (90%) in predicting non-infected gastric mucosa. RAC-positive pattern provided excellent PPV (96.3%) for exclusion of *H. pylori* infection which was similar to prior studies [15, 25].

Image-enhanced endoscopy has been integrated into everyday practice to augment endoscopic diagnosis of gastric precancerous lesions and *H. pylori* infection. This study displayed numerically higher sensitivities of M-NBI and LCI than comprehensive findings on WLI which were similar to previous reports [9, 20]. However, only M-BLI provided significantly higher sensitivity than comprehensive findings on WLI. LCI can improve sensitivity in *H. pylori* diagnosis by emphasizing modest color differences of gastric mucosa and consequently enhance visibility of mucosal redness [9, 26]. Our study demonstrated that LCI provided 30.4% and 57.1% sensitivity improvement by increasing detection of diffuse redness and spotty redness, respectively. This is consistent with the previous review mentioning LCI as a screening tool for detection of inflamed mucosa especially in wide luminal organ such as stomach [11]. On the contrary, M-BLI and M-NBI promote diagnostic ability by assessing pit and vascular patterns at magnified close-up view [11]. M-BLI yielded maximal sensitivity for *H. pylori* diagnosis in our study, followed by M-NBI. However, there was no difference between M-BLI and M-NBI in *H. pylori* detection which was similar to the previous report [12]. One fifth of *H. pylori*-infected patients had endoscopically normal gastric mucosa on WLI, but most of them had endoscopic *H. pylori* detection while using M-BLI or M-NBI. This could be because more severe pit pattern change was correlated with higher histological severity of *H. pylori*-related gastritis [17]. Therefore, early minor change of mucosal pattern detected on magnifying IEE might be presented before it could be noticed by WLI. This study added value of M-BLI and M-NBI in the diagnosis of *H. pylori* infection in dyspeptic patients with normal-appearing gastric mucosa.

Other studies using IEE for diagnosis of *H. pylori* infection demonstrated the highest sensitivities by BLI or NBI (90–98%) followed by LCI (84–95%), whereas WLI had a wide range of relatively lower sensitivities (67–92%) compared with IEE as shown in Supplementary Table 1. Most studies were from Asian countries, especially from Japan. Majority of studies compared diagnostic performance of IEE to WLI except for the study by Özgür et al. comparing NBI to gold standard tests and Tahara et al. comparing BLI to NBI [9, 12, 20, 26–29]. Our research was the only one to compare all 4 endoscopic methods and demonstrated comparable sensitivities and specificities of WLI and IEE to other studies. This study revealed excellent sensitivities of BLI (95%) and NBI (92.5%) in *H. pylori* detection and demonstrated higher specificities and PPV of enlarged gastric folds and antral nodularity on WLI for predicting *H. pylori* infection than previous studies as demonstrated in Supplementary Table 2 [7, 21, 24]. According to excellent diagnostic performance, IEE might be an alternative option for rapid endoscopic diagnosis of *H. pylori* infection in the near future.

In conclusion, the sensitivity of enlarged or elongated pits with obscure SECNs or dense fine irregular vessels on magnifying BLI for diagnosis of *H. pylori* infection was significantly higher than comprehensive findings on WLI. Enlarged gastric folds and antral nodularity could be reliable predictors for *H. pylori* infection, while fundic gland polyps and red streak could be important endoscopic findings for *H. pylori*-negative mucosa.

### Electronic supplementary material

Below is the link to the electronic supplementary material.

Supplementary Material 1

Supplementary Material 2

## Data Availability

The datasets generated and analysed during the current study are not publicly available due to privacy but are available from the corresponding author on reasonable request.
