# Supplementary material for: Simultaneous detection of Helicobacter pylori infection comparing between white light and image-enhanced endoscopy
Source: BMC Gastroenterol. 2024 Jan 26;24:46. doi: 10.1186/s12876-024-03132-y (PMC10811817; doi:10.1186/s12876-024-03132-y)
Supplement: Supplementary file 1 — Supplementary Material 1 [file 12876_2024_3132_MOESM1_ESM.docx]

**Supplementary Table 1.** Review of studies using IEE for diagnosis of *H. pylori* infection

| **Studies, year** | **Country** | **Total patients** | ***H. pylori* prevalence** | **Diagnostic methods for *H. pylori* infection** | **WLI**  **Sens/Spec** | **LCI**  **Sens/Spec** | **BLI**  **Sens/Spec** | **NBI**  **Sens/Spec** |
| --- | --- | --- | --- | --- | --- | --- | --- | --- |
| This study, 2023 | Thailand | 100 | 40% | RUT, histology, culture | 80%/71.7% | 90%/70% | 95%/80% | 92.5%/86.7% |
| Cho et al.,[[27](#_ENREF_27)] 2021 | South Korea | 254 | 64.2% | RUT, PCR | 92%/95.6% | - | - | 96.3%/95.6% |
| Tahara et al.,[[20](#_ENREF_20)] 2019 | Japan | 163 | 55.8% | Histology, UBT, serology | (A) 72.2%/75.5%*  (B) 86.6%/57.3%* | - | - | (A) 96.9%/93.6%*  (B) 92.8%/93.6%* |
| Özgür et al.,[[28](#_ENREF_28)] 2015 | Turkey | 165 | 33.9% | Histology, culture | - | - | - | 92.9%/62.4% |
| Wang et al.,[[29](#_ENREF_29)] 2019 | China | 103 | 26.2% | RUT, histology | 67%/79.96%** | 85.4%/79.7%** | - | - |
| Ono et al.,[[26](#_ENREF_26)] 2020 | Japan | 127 | 50.4% | UBT, serology | 84.4%/74.6% | 84.4%/88.9% | - | - |
| Dohi et al.,[[9](#_ENREF_9)] 2016 | Japan | 60 | 50% | RUT, histology, serology, UBT | 81.7%/66.7% | 93.3%/78.3% | - | - |
| Tahara et al.,[[12](#_ENREF_12)] 2017 | Japan | 225 | 59.1% | UBT, histology, serology | - | - | 98%/92% | 97%/81% |

* Results were separated according to endoscopist A or B.

** Evaluation at corpus
