# Supplementary material for: Simultaneous detection of Helicobacter pylori infection comparing between white light and image-enhanced endoscopy
Source: BMC Gastroenterol. 2024 Jan 26;24:46. doi: 10.1186/s12876-024-03132-y (PMC10811817; doi:10.1186/s12876-024-03132-y)
Supplement: Supplementary file 2 — Supplementary Material 2 [file 12876_2024_3132_MOESM2_ESM.docx]

**Supplementary Table 2.** Diagnostic performance of endoscopic features for diagnosis of *H. pylori* infection by WLI

| **Endoscopic findings** | **Studies, year** | **Country** | **Total patients** | ***H. pylori* prevalence** | **Sensitivity** | **Specificity** | **PPV** | **NPV** |
| --- | --- | --- | --- | --- | --- | --- | --- | --- |
| **Diagnostic performance to predict *H. pylori*-positive status by WLI** | | | | | | | | |
| Enlarged gastric folds | This study, 2023 | Thailand | 100 | 40% | 12.5% | 100% | 100% | 63.2% |
|  | Yoshii et al., 2020 | Japan | 494 | 15.8% | 23.1% | 96.6% | 56.2% | 87.0% |
|  | Mao et al., 2016 | China | 256 | 44.1% | 60.2% | 92.3% | 86.0% | 74.6% |
|  | Kato et al., 2013 | Japan | 275 | 59.6% | 58.5% | 79.5% | 76.9% | 62.2% |
| Antral nodularity | This study, 2023 | Thailand | 100 | 40% | 10% | 100% | 100% | 62.5% |
|  | Yoshii et al., 2020 | Japan | 494 | 15.8% | 6.4% | 98.3% | 41.7% | 84.9% |
|  | Kato et al., 2013 | Japan | 275 | 59.6% | 58.5% | 79.5% | 76.9% | 62.2% |
| **Diagnostic performance to predict *H. pylori*-negative status by WLI** | | | | | | | | |
| Fundic gland polyps | This study, 2023 | Thailand | 100 | 40% | 16.7% | 100% | 100% | 55.6% |
|  | Yoshii et al., 2020 | Japan | 494 | 15.8% | 34.9% | 93.4% | 90.2% | 45.5% |
|  | Mao et al., 2016 | China | 256 | 44.1% | 14.7% | 95.6% | 80.8% | 47% |
|  | Kato et al., 2013 | Japan | 275 | 59.6% | 24% | 97.9% | 91.2% | 59.2% |
| Red streak | This study, 2023 | Thailand | 100 | 40% | 8.3% | 100% | 100% | 42.1% |
|  | Yoshii et al., 2020 | Japan | 494 | 15.8% | 32.7% | 90.7% | 85.8% | 43.9% |
|  | Mao et al., 2016 | China | 256 | 44.1% | 3.5% | 100% | 100% | 45% |
|  | Kato et al., 2013 | Japan | 275 | 59.6% | 0.9% | 84.4% | 96.2% | 45.3% |
| RAC | This study, 2023 | Thailand | 100 | 40% | 86.7% | 95% | 96.3% | 82.6% |
|  | Yoshii et al., 2020 | Japan | 494 | 15.8% | 89.1% | 79.8% | 87.9% | 81.6% |
|  | Kato et al., 2013 | Japan | 275 | 59.6% | 92.3% | 93.6% | 87% | 66.8% |

NPV = Negative predictive value, PPV = Positive predictive value, RAC = Regular arrangement of collecting venules, WLI = White light imaging
